# Knowing to Ask and Feeling Safe to Tell - Understanding the Influences of HCP-Patient Interactions in Cancer Care for LGBTQ+ Children and Young People

**DOI:** 10.3389/fonc.2022.891874

**Published:** 2022-06-24

**Authors:** Tamsin Gannon, Bob Phillips, Daniel Saunders, Alison May Berner

**Affiliations:** ^1^ Paediatric and Teenage and Young Adult Oncology, The Royal Marsden NHS Foundation Trust, Sutton, United Kingdom; ^2^ Paediatric and Teenage and Young Adult (TYA) Oncology, Leeds Children’s Hospital, Leeds, United Kingdom; ^3^ Clinical Oncology, The Christie NHS Foundation Trust, Manchester, United Kingdom; ^4^ Barts Cancer Institute, Queen Mary University of London, London, United Kingdom; ^5^ Gender Identity Clinic, Tavistock and Portman NHS Foundation Trust, London, United Kingdom

**Keywords:** LGBTQ+, sexual orientation, gender identity, healthcare professional attitudes, healthcare professional knowledge, healthcare professional behaviour change, paediatric oncology, teenage and young adult cancer

## Abstract

**Background:**

Lesbian, gay, bisexual, transgender, queer or questioning (LGBTQ+) people experience healthcare inequalities in cancer care. Previous studies have focused on knowledge, attitudes and behaviours of healthcare professionals (HCPs) treating adults with cancer and how these contribute to inequalities. To date, no research has focused on HCPs treating LGBTQ+ children and adolescents with cancer in the UK. This is important given that this group may be at a critical time for exploring their gender identity and sexual orientation, whilst also facing a cancer diagnosis. We aimed to explore the knowledge, attitudes and behaviours of paediatric, teenage and young adult oncology HCPs treating LGBTQ+ patients in the UK.

**Methods:**

We carried out semi-structured interviews with 8 HCPs in paediatric, teenage and young adult (TYA) oncology from the Royal Marsden NHS Foundation Trust. Eight questions were asked, which centred around participants’ knowledge, attitudes and behaviours regarding management of LGBTQ+ patients in oncology. Interview transcripts were analysed by inductive thematic analysis.

**Results:**

We identified 10 themes, including novel themes (how HCPs acquire knowledge and expectations of a ‘third party’ to be the expert) which may underlie previously observed trends in knowledge, attitudes and behaviours of HCPs. We highlight other themes and HCP concerns specific to care of LGBTQ+ patients in paediatrics (influence of the parental-carer dynamic, concerns around patient age and development as a barrier to disclosure) which require further research. We found evidence of the interrelatedness of HCP knowledge, attitudes and behaviours and the ability of these elements to positively influence each other. We mapped our themes across these elements to form a new suggested framework for improving HCP-patient interactions in LGBTQ+ Cancer Care. We found a need both for individual HCP education and organisational change, with creation of a culture of psychological safety to improve patient care.

**Conclusion:**

Knowledge, attitudes and behaviours of HCPs are closely interdependent when providing care to young LGBTQ+ patients with cancer. The authors suggest that future efforts to improve care of these patients address this complexity by spanning the domains of our suggested framework. Whilst HCP education is essential, change must also occur at an organisational level.

## 1 Introduction

Sexual minorities are those who identify with any sexual orientation (SO) other than heterosexual, including gay, lesbian, bisexual, asexual, pansexual. It also includes those questioning their SO. Gender minorities are those whose gender identity (GI) is different from the sex they were assigned at birth. This includes a range of identities including transgender and gender diverse which are also umbrella terms. Here we will use the acronym LGBTQ+ (lesbian, gay, bisexual, transgender, queer or questioning) to encompass sexual and gender minority communities.

Estimates from western countries suggest that 2.7%-7.1% of people identify as LGBTQ+ (1–3) and this is rising due to increased disclosure as a result of changing society attitudes (4). In 2016, sexual and gender minorities (SGMs) were identified as a health disparity population in research by the National Institute for Health (5) and a recent UK Government Equalities Office review reported an urgent need to address the ‘inequality in service provision and delivery, particularly in health’ for this group (6).

SGM people experience minority stress and poorer health outcomes compared to cisgender, heterosexual people. Challenges are worse for those who identify in more than one minority group (7). Intersectionality, is the term used to describe this interconnected nature of social categories that creates overlap of discrimination.

LGBTQ+ populations experience myriad inequalities across healthcare (8–12) with poorer experience, worse health outcomes and being more likely to access mental health services (likely as a result of the minority stress). They cite a lack of healthcare professional (HCP) knowledge on specific LGBTQ+ needs, experiences of heteronormativity and discrimination (6).

Cancer is a particular area of unmet need. LGBTQ+ adults experience disparities across the continuum of cancer care from screening, through diagnosis and management, to end of life care (8, 13–17). They are at higher risk of some cancers due to higher rates of risk behaviours (7). They are more likely to delay initial presentation to healthcare due to prior discrimination or negative experiences. They report lower satisfaction with cancer treatment, higher rates of psychological distress in survivorship and poorer health outcomes (7). A major concern for LGBTQ+ cancer patients is whether to disclose their GI and/or SO, considering if this will result in discrimination and poor care (18).

In 2017, the American Society of Clinical Oncology published a statement on reducing cancer health disparities for this population (19). In 2021, a statement from the Joint Collegiate Council for Oncology made a set of commitments signed by organisations across cancer care in the UK, which included greater research and improved education on LGBTQ+ cancer care (20).

There are features unique to cancer care in children and adolescents, such as increased prominence of the carer-patient relationship, that may affect interactions with HCPs and a recent study found that young LGBTQ+ people with cancer experienced higher distress and confirmed they were less likely to disclose their SO or GI than older adults (21). However, there remains a relative lack of research into healthcare experiences of LGBTQ+ youth specifically, and much of our current knowledge is based on research on LGBTQ+ adult health. In 2019, Australian researchers published a call to action aimed at reducing the research gap in Teenage and Young Adult (TYA) cancer care. They categorised LGBTQ+ young people with cancer as at-risk group due to the psychosocial and systemic vulnerabilities experienced by this group in healthcare (7). Common challenges for TYAs through their cancer journey include body image concerns, mental health and the loss of independence. The impact of questioning ones SO or GI through their cancer journey is often overlooked (7).

Young people aged 16 to 24 years are the most likely age group to identify as LGB with 4% belonging to a sexual minority group (3). There is no robust UK data on younger age groups but 9.5% of those aged 13-17 years from the USA identify as LGB (22).Population estimates on trans youth in the UK are lacking, but international data suggest that 1.2% to 2.7% of children and adolescents identify as transgender (23). A freedom of information request found that as of 31st December 2019 there were 4220 under 18s on the waiting list for GI services (24).

Disclosure to an HCP may also be a greater challenge for TYAs who may not want or be able to disclose to their family/friends, who may not have the language or understanding of their emotions to be able to discuss their emerging SO or GI (21). Disclosure is made even more difficult in adolescent care due to the family centred approach if the reason for non-disclosure is family or carer presence. In a study of 102 LGBTQ+ young people, 75% of participants reported they did not disclose as they did not want to discuss SO in front of parents/carers (25). Previous studies also suggest paediatricians do not address SO or GI and a study on LGBTQ+ adolescents identified only 35% had disclosed their identity to their healthcare professional whilst 64% would have communicated this information if they were asked (26). Research shows disclosure of LGBTQ+ identity has a positive impact on patients’ health experience and improved well-being (27). LGBTQ+ youth expressed a desire for more open discussions regarding their sexual and emotional health (28).

Several studies have focussed on the attitudes and knowledge of HCPs treating LGBTQ+ adults with cancer. These are mainly from the USA, one from the UK and one from Australia (29–36). Some focused solely on individual HCP groups such as doctors (29, 32, 33), oncology advanced nurse practitioners (31), radiotherapists (35), and a few have examined the broader multi-disciplinary team (30, 34, 36) reflecting the multi-disciplinary approach of cancer care.

Despite the heterogeneity in location and HCP surveyed, there has been a consistent finding of a paucity of self-perceived knowledge in the specific healthcare needs of LGBTQ+ patients accessing cancer services, and a desire for greater education. In those studies where knowledge was tested, the percentage of participants that could correctly answer all questions ranged between 3% and 50% (30, 34, 36). Across studies, it was felt knowledge of GI, sex assigned at birth and intersex variations were more important than SO to provide the best cancer care (32, 33) and yet there tended to be the least confidence in knowledge on care of gender diverse patients (29, 33, 34), suggesting this attitude did not prompt knowledge acquisition.

Non-physicians tended to be more confident than physicians in their knowledge and also tended to have a greater interest for education on LGBTQ+ health (34). Further, a higher percentage of nurses and allied health professionals felt this topic should be mandatory compared to medical practitioners (34) These differences of opinion may be the result from differing perceptions around the relevance of this topic to one’s job role. Other reasons cited by HCPs for their low knowledge of LGBTQ+ health were competing clinical and educational demands and lack of evidence-based guidelines (32).

Across studies the majority of participants regardless of profession reported feeling comfortable treating LGBTQ+ patients (30–32). However, comfort did not appear to correlate with knowledge overall (30) or to translate into behaviours of active enquiry around LGBTQ+ identity (30) though in UK oncologists it resulted in a greater confidence in overall communication (29).

With regards to specific behaviours, only 2-48% of HCPs across studies explicitly encouraged disclosure of LGBTQ+ identity (29, 30, 36). Assumptions about SO and GI were high (29, 30, 32, 34). However, as these studies have been mostly quantitative, they cannot fully capture relationships between these behaviours and underlying knowledge and attitudes. The qualitative interview-based study by Sutter *et al.* provided more detail and aided current understanding of this topic in adult cancer care. HCPs stated LGBTQ+ concerns may be neglected because ‘survival took precedence’ and due to HCP fears around using the correct language and making assumptions (32).

To-date there have been no published studies solely on the knowledge, attitudes and behaviours of HCPs in Paediatric Oncology. Ussher *et al.* include HCPs caring for Paediatric and TYA patients but responses for this subgroup were not analysed (34). In Sutter *et al.* adolescent cancer care was also described and the benefit of having clinical expertise in LGBTQ+ health was highlighted when an oncologist reported having a specialist from a gender dysphoria clinic was invaluable in assisting them care for a transgender adolescent patient. Effects of family conflict were also raised and the importance of providing a supportive place to disclose SO and GI in hospital if it was not safe to do so at home (32).

In the UK, the doctors delivering cancer care for children, teenagers and young people are mainly paediatricians. In a Canadian study, knowledge regarding LGBTQ+ issues were limited amongst paediatricians (37) and LGBTQ+ young people describe a lack of LGBT-tailored knowledge/support when accessing healthcare (38, 39). However, oncology care involves a multidisciplinary team of HCPs from different disciplines and there have been no studies specific to HCPs delivering paediatric and TYA cancer care in the UK. LGBTQ+ healthcare education in UK medical schools and in the undergraduate curriculum of other HCPs is variable and poor, with a few notable exceptions of good practice (40, 41). Rarely is LGBTQ+ health discussed specifically with curriculum documents (42).

We therefore set out to explore the knowledge, attitudes and behaviours of paediatric oncology HCPs treating paediatric, teenage and young adult LGBTQ+ patients in the UK, and to do so qualitatively, to more deeply explore reasons behind the findings observed in previous studies of HCPs treating adults. We then aim to use our findings to better define the educational need of HCPs treating young LGBTQ+ patients with cancer and make recommendations around its delivery.

## 2 Materials and Methods

### 2.1 Ethics Approval

The study was approved by the Royal Marsden NHS Foundation Trust and the Institute of Cancer Research Ethics committee (Ref No: SE 1132).

### 2.2 Recruitment

Recruitment was *via* an advertising email sent to all HCPs working in Paediatric Oncology and the project was advertised at handovers/multi-disciplinary meetings.

Participants needed to be; 1) working at Royal Marsden Hospital NHS Foundation Trust, 2) a paediatric oncologist or haematologist, clinical nurse specialist, nurse practitioner, psychologist or psychology assistant, allied health professionals or play therapists 3) caring for paediatric, teenage or young adults with cancer currently and for a minimum of 6 months prior to the interview. All participants provided written informed consent.

### 2.3 Participants

Discussion of how many participants from each HCP group was decided amongst the study team. It was decided to review whether there was thematic saturation once at least 8 participants had been interviewed.

Participants comprised of 3 Paediatric Oncologists, 2 Clinical Nurse Specialists, 1 Speech and Language Therapist, 1 Occupational Therapist and 1 Psychologist. They were aged between 24-59 years (median 39 years). All participants identified as female which correlates with the high percentage of women in Paediatrics (there are more female consultants than male and 74% of trainees are female) (43). Participants had been in their role for a median of 7 years (range 18 months to 23 years). All participants worked with children, teenagers and young adults and none identified as LGBTQ+. We define children as those aged under 13 years, teenagers aged 13-18 years and young adults 19–25 years. One participant did not consent to their demographic details being published. Three participants had attended a recent education session by a Paediatric Oncologist during Pride about LGBTQ+ history.

### 2.4 Setting

Interviewed staff were based at the Royal Marsden Hospital based in Sutton, England. The Royal Marsden is a tertiary oncology centre, a leader in the field of cancer treatment and research and is expected to be ahead of other centres regarding education and training such as LGBTQ+ cancer care. Patients have access to a multidisciplinary team which includes Paediatric Oncologists, Paediatric Haematologists, Adult Haematologists, Advanced Nurse Practitioners, Clinical Nurse Specialists, Allied health professionals, Psychologists etc.

Data for this study was collected in November 2021 post COVID-19 pandemic. The NHS Rainbow badge had been introduced several months prior to interviews in early 2021.

### 2.5 Interviews

Virtual semi-structured interviews (duration range: 30-60 minutes) were carried out *via* Microsoft Teams. Interviews were recorded and stored *via* Microsoft Teams and automated transcription was used. Participants were advised to carry out the interviews in a private space. All interviews were carried out by TG. Eight questions were asked which centred around participants’ knowledge, attitudes and behaviours regarding management of LGBTQ+ patients in oncology including how to manage a hypothetical scenario.

### 2.6 Patient and Public Involvement

Development of our interview questions were guided by patient/public involvement groups. We attended two focus groups. The first was run virtually by the Teenage Cancer Trust charity and comprised 2 participants, both aged 22 years old, both on active treatment for cancer and who stated they were part of the LGBTQ+ community. The second group was the Youth Forum run at The Royal Marsden hospital. There were 7 participants in this group, aged between 18-24 years who were either on active treatment, in remission or post treatment. 5 identified as part of a minority group.

### 2.7 Data Analysis

All interviews were re-watched, and automated transcripts were anonymised and edited by TG. Transcripts were then read and re-read. We conducted a thematic analysis of interview responses using an inductive, experiential and critical realist approach in line with previously published recommendations (44). TG and AMB carried out data familiarisation separately. Initial coding was carried out by TG with separate checking and additional coding by AMB. Codes were then reviewed with an inductive approach to group similar codes and identify themes that may be relevant to the overarching research question and aims. During coding of the last 2 interviews few new codes were created and therefore no new patterns/themes were found in the data therefore it was felt we had reached thematic saturation. Themes, their evidence and their interrelatedness were discussed among the whole study team to develop the suggested framework.

### 2.8 Reflexivity Statements

The authors acknowledge that the approach they bring as researchers will influence their approach to research, and specifically the themes that are identified and developed through the analysis. For clarity, as AMB and TG worked with the primary data, they here provide reflexivity statements as to how they approach the work.

Author AMB approaches this study through the lens of both a LGBTQ+ health researcher and a cancer physician, as well as a sexual minority cisgender woman. Author TG approaches this study through the lens of a trainee paediatrician as well as an ethnic minority who is interested in health equality and equity. As a cisgender woman she is aware she has not experienced the discrimination members of the LGBTQ+ community may face. However, as a member of a minority group is interested in intersectionality in healthcare. Both researchers acknowledge an implicit bias that comes from their knowledge of the existing literature on this topic and from the assumptions of a need for training of HCPs on this topic that has driven the research question.

## 3 Results

Dual coding produced 191 tentative codes, which were rationalised to 151 final codes. These produced 10 themes (**Table 1**) following iterative discussion and rationalisation.

**Table 1:** Themes identified through analysis of HCP interviews.

1. Benefits and harms of disclosure and non-disclosure
2. Barriers and facilitators of disclosure and enquiry
3. Lack of confidence in knowledge of LGBTQ+ cancer care
4. Knowledge of appropriate language
5. How knowledge of LGBTQ+ cancer is acquired
6. The ‘third party’ as the expert on the topic of LGBTQ+ cancer care.
7. Parental-carer and patient dynamic
8. The patient as an individual
9. Discussing sex as part of cancer care
10. Visible LGBTQ+ affirming materials

SO, sexual orientation; GI, gender identity; HCPs, healthcare professionals.

### 3.1 Benefits and Harms of Disclosure and *Non-Disclosure*

Disclosure of LGBTQ+ identity was a common recurring theme throughout all interviews. Disclosure is at the core of this topic as without it many clinicians may assume heterosexuality and cisgender identity, and be unable to tailor their care for LGBTQ+ patients. Evidence reported by LGBTQ+ TYAs highlighting their negative experience of healthcare included a lack of active enquiry by HCPs regarding their SO as a negative factor (7). Inclusive discussion of SO by HCPs (as opposed to heteronormative assumptions) has been linked to positive health and mental health (45, 46). Previous studies in adult patients have identified the perception that disclosure improves overall care and improves trust with the HCP (47) but also that it entails risks including discrimination and unequal care (48, 49).

Participants were aware of some of the previously reported benefits of encouraging disclosure of LGBTQ+ identity by patients. These included improvement of trust in the HCP-patient relationship: ‘if they feel able to do that (share their SO/GI), that can foster the sense of trust between the clinician and the patient’ and provision of better overall healthcare by tailored support to their needs: ‘if we don’t know a patient identifies as LGBTQ+ we don’t know a lot of their life perspective and we don’t know about a really important part of their identity, so it’s going to be more difficult to meet their needs adequately.’

However, participants highlighted many more specific situations where this was of particular relevance, such as discussion of the benefits of hormone replacement therapy could have for the patient’s sex life; ‘when we had a conversation about sexuality and that hormones helped your vagina become moist and cushioned and that might help sexual pleasure … they started taking their HRT.’

Participants also felt that there were more unique benefits of knowing a patient’s GI was different from their sex assigned at birth. One such reason was so that the patient can be correctly identified and addressed accordingly: ‘If it’s important that we identify the patient as they want to be, then we should know’ and ‘it might help, I’m thinking in terms of how people use their pronouns’. There was also acknowledgement of how trans status may impact the future health risks for the patient ‘if we’re specifically talking about something that involves sexual organs … if someone identifies as male, but has a womb and I need to talk about the risk.’

Examples were raised where a lack of acknowledgement of someone’s SO or GI could cause harm such as the insensitive discussion of contraception, and a gender diverse young person not wanting to exercise due to body dysphoria. Another participant described how the consequences of cancer treatment for gendered body development needed to acknowledge the patients’ feelings towards their gender to be sensitive and support the patient to engage with healthcare.

Previous studies involving both HCPs and patients have been less specific about the apparent health benefits to care. Much literature discusses the relevance in terms of patient-provider relationship and of risk of cancer according to bodily organs and behaviours in adults (27, 50–52), but the perceived benefits here relate to the ongoing health and experience of the young person living with and beyond cancer, and deserve special attention in the education.

While participants recognised the relevance of patients’ SO and GI to their psychological needs due to the likelihood of poorer mental health: ‘missing what may be contributing to mental health problems and suicidal ideation,’ some participants identified this as the sole harm of non-disclosure ‘the harm is if they’re having psychological difficulty, and it’s something that we could help with’. While poorer mental health outcomes in LGBTQ+ young people are well recognised (53, 54), this view overlooks other important aspects to care and perhaps even indicates a level of stigma from the healthcare clinician that LGBTQ+ identity is in itself a mental health concern. There was agreement amongst participants that exploring LGBTQ+ identity at the same time as having cancer treatment may cause additional stress which is important for HCPs to acknowledge: ‘just thinking of like the wider picture that we’re kind of here about the cancer diagnosis and that maybe the patient has a lot of other thoughts going on at the moment whether they were planning a transition.’

Exclusion of chosen family was a key harm identified, with one participant commenting: ‘maybe not understanding partnerships that might want to be involved in the care or you know involved in providing some sort of support’ as a harm of non-disclosure. Participants discussed the detrimental impact of assumptions about the relationship of the person that a patient is bringing to a consultation, which is well recognised in adults (48).

Despite much literature detailing the perceived risks of stigma and discrimination from disclosure of SO/GI (27, 36, 45, 46, 48, 55–58) this was recognised by only two participants: ‘you just have to be careful that knowledge doesn’t allow the opportunity for prejudice’, ‘you’ll probably find a range of attitudes within the health care team … sometimes people unconscious behaviour can have an impact on our patients.’ Multiple other participants commented that this was not an issue they had witnessed in their careers: ‘I’ve never really come across sexuality being an issue within a healthcare setting … I’ve never personally come across it affecting any decisions or making anyone feel uncomfortable’. This may reflect the fact that direct discrimination often does not take place in the presence of other HCPs or that it is indirect and may not be viewed as such by HCPs who lack cultural competence. A recent UK study looking at HCP care of LGBTQ+ youth during the pandemic noted the challenge of managing prejudice within teams as one of its themes, with one participant stating this was “silence more than with nasty comments” (59).

### 3.2 Barriers and Facilitators of Enquiry by HCP/Disclosure From Patient

While existing literature has been less specific as to the benefits of disclosure of SO/GI, much more exists detailing its barriers and facilitators. HCP behaviours that cause LGBTQ+ patients to hesitate when disclosing identity include perceived HCP discomfort post disclosure, failing to answer LGBTQ+ specific care questions adequately, using inappropriate language, stereotyping and presumptions of incorrect relationships such as friend or relative between the patient and their partner (7).

Brooks *et al.* carried out a systematic review of literature across healthcare and found four broad themes: “the moment of disclosure”, “the expected outcome of disclosure”, “the healthcare professional’, and “the environment or setting of disclosure” (48). Banerjee *et al.* looked at this area specifically within oncology by surveying 1,253 HCPs in the USA using open ended questions on how HCPs encouraged disclosure, communication challenges, structural/system challenges and their own recommendations on the management of LGBTQ+ patients (36).

These broad categories are mirrored in some of our own findings.

#### 3.2.1 Expected Outcomes of Disclosure

A key apparent barrier for enquiry about LGBTQ+ identity was not being aware of its general relevance to the patient’s healthcare, and the benefits and harms discussed above, as well as our later themes around knowledge. Most participants felt they needed a specific reason to ask about LGBTQ+ identity: ‘I suppose if we’re specifically talking about something that involves sexual organs that might be important to share.’ Brooks and colleagues described the theme of expected outcome of disclosure as relevant to the patient’s choice to disclosure (48) but here we also see it relevant to the HCPs willingness to enquire. If they see no difference in the outcome, they will not enquire, or at least place it lower on the HCP agenda.

This led to views that SO/GI was only relevant to the consultation if it was particularly relevant to the patient: ‘I feel like I don’t need to know unless you want to tell.’ Most HCPs interviewed also thought that if LGBTQ+ identity was important to the patient they would bring it up, which is in contrast to recent studies that suggest LGBTQ+ young people may not disclose SO or GI so readily in this context. (21)

In some cases, these attitudes appeared to stem also from a place of respect for the patient’s wishes: ‘it’s up to the patient if they want to disclose how they identify themselves’ and the fact that teenagers in particular may find this information sensitive ‘sexuality during your teen years can be something that is private to yourself’. All participants felt patients should only disclose if they feel comfortable to do so and disclosure should not be mandatory: ‘I just am mindful I wouldn’t want people to feel like they would have to share it.’ Whilst this is true, over-emphasis on the assumptions that patients wish this information to be private and will disclose, represent barriers to disclosure and a risk to the patient in accessing optimal care.

One participant did comment on the patient’s expectations of disclosure and how this might underlie their reasons for doing so: ‘is it that they’re telling me this because they have been hurt, are they telling me this because they’re asking for help? Are they telling me this because something negative has happened or are they telling me because they’re very comfortable in their GI?’ Cultural humility (“ability to maintain an interpersonal stance that is other-oriented (or open to the other) in relation to aspects of cultural identity that are most important to the person”) (60) is needed to understand the range of emotions associated with disclosure and something HCPs can develop to facilitate disclosure and provide more tailored care (61).

Other previously noted facilitators (48) that relate to the patients expected outcomes following disclosure observed in our study include respect of confidentiality: ‘it’s about reassuring that young person that, unless they’re at harm or someone else is at harm, than it does, stay private & really explicitly agreeing with that patient who else is allowed the privilege of that information’. SO and GI documentation on a computer system to avoid repeated disclosure: ‘sometimes people say. I’m really tired of coming out all the time it’s quite exhausting having to retell my story time and time again, so actually having a really clear documentation on the electronic patient record (or) shared with the team *via* email can often be a relief to a patient’.

#### 3.2.2 HCP Factors

The work of both Brooks *et al.* and Banerjee *et al.* separates those facilitators and barriers that relate directly to the HCP (including their communication), the setting of disclosure and context and the overall healthcare system (36, 48). Our study found factors within each of these realms that affected disclosure. Whilst some of these were previously noted they showed greater prominence in our work. For example, while low HCP confidence has often been noted in this literature (29–36), we found that a commonly cited barrier for enquiry by HCPs was overt fear. This included fear of: ‘getting it wrong’, ‘embarrassing themselves’ and ‘making (patients) feel uncomfortable’. Some of these were also highlighted in the aforementioned study by Banerjee *et al.*

HCPs also spoke of a culture where questions regarding SO/GI are only being asked secondary to assumptions that have been made about the patient, especially those based on appearances. HCP are fearful to voice these assumptions and cause offence: ‘we’re worried about falling into stereotypes…”.

Naming the barriers as specific ‘fears’ better allows these to be tackled head on in efforts to improve confidence and overall care. For example, increased awareness and dialogue amongst colleagues was found to be a facilitator for disclosure conversations. One participant noted that one such discussion ‘brought down all barriers to be able to talk about [SO/GI] between staff because it was something that became very comfortable following that’. This also shows that while a barrier may be specific to the HCP, overcoming it may not be down to the individual HCP alone.

Another HCP-specific factor is the belief that equal care is equitable care which again feeds the participant’s view that LGBTQ+ identity was not important to cancer care: ‘I don’t treat people differently. You know, if they’re a different race or … it makes no difference to me. From my point of view, it doesn’t really change how I treat the person.’ This view may result in a lack of insight into potential for unconscious bias and fails to acknowledge the unique healthcare needs of some minority groups. Such an approach was noted by Ussher *et al. (* 22) who named it an ‘egalitarian’ approach.

Other participants felt a conscious bias by other HCPs who may hold anti-LGBTQ+ beliefs were a barrier to broaching the topic: ‘there might be some people who would treat them differently because of their own belief system’. While fear of discrimination and perception of HCP prejudice have both been noted as barriers for disclosure (46, 48). This view may mean that the detrimental effect of prejudice is therefore more far reaching as it indirectly impacts access to tailored care through reduced enquiry by other HCPs who do not themselves hold prejudice.

By contrast, a facilitator of disclosure was the attitude that all HCPs should be taking an active role into enquiry rather than waiting for the patient to disclose: ‘I think that healthcare professionals can be taking responsibility for asking people if its ok to have a conversation about SO/GI and for that to be done with everybody.’

The need to consider the HCP experience related to LGBTQ+ identity was raised by some participants. This includes whether they themselves identify as LGBTQ+, as well as interactions with friends or family who are LGBTQ+. Previous studies have described this as a facilitator (48) but depending on the HCP experience can lead to personal biases, which was noted by one of our participants.

Most participants felt it was important for the HCP to have developed a good relationship and rapport with the patient before disclosure: ‘I think that’s probably the most important thing is a kind of a trusting relationship that develop where people can speak about it if they wish’. It was also noted that the type of relationship formed between HCP and patient was more a facilitator of disclosure compared to the duration of relationship: ‘there was a little bit of a relationship there, a couple of sessions in, not like weeks and weeks or months like you know, we see patients for a very long time sometimes.’ Both short and long duration of relationship have previously been found to be facilitators (48).

HCPs from different professions may prioritise information on patient’s SO/GI differently depending on how it relates to the sort of care they provide. Placing this information higher on a clinician agenda is likely to encourage greater disclosure. One allied health professionals who described treating numerous LGBTQ+ patients in their short career disclosed: ‘in my experience, it’s actually come up very casually’ in conversation compared to an oncologist who believed they ‘haven’t looked after anybody who was gay’. It was felt that nurses also place this higher on their agenda than doctors: ‘TYA nurses, for example, are quite tuned into it. Maybe the clinicians less so probably. I guess that might vary between different clinicians as to how comfortable they are’.

The data also suggested that knowledge and awareness of the disadvantage and discrimination the LGBTQ+ community faces may result in this information being higher on the HCP agenda: ‘I think the evidence would tell us that people who identify as being in the LGBTQ+ community face social disadvantage … if you don’t know that your patient has had that in their background you can’t support them and be sensitive to their needs.’

#### 3.2.3 Consultation Skills

Our participants described many of the same aspects of the HCP-patient consultation that were noted as facilitators or barriers to disclosure in previous work (34, 36, 48) under themes that cover communication skills, setting and environment. These included open questioning style, consultation space, time allocated for the consultation and who is present during the consultation. Although many of these practices are good practice for consultations discussing sensitive issues more broadly, they are of particular value when approaching topics that may be sensitive for the patient, and so it is crucial to reinforce their necessity.

One participant facilitated disclosure by providing patients with the reasoning as to why these personal questions were being asked: ‘I give the rationale … I try to allow people to understand where I’m coming from and why it’s important that I do this … I want to get to know who they are’. If patients are aware that these questions are being asked so that HCPs can tailor their healthcare in order to improve it, they may be more willing to discuss other parts of their life. This technique has been described previously but we note its reliance on the HCPs knowledge of the importance of enquiry about SO/GI and its relevance to healthcare, demonstrating the interrelatedness of these two concepts.

#### 3.2.4 Structural Factors

Participants noted structural barriers to providing good care of LGBTQ+ people overall (such as encouraging disclosure) within the UK health system.

Participants felt changing the attitude around this topic was needed: ‘it’s just got to become more mainstream.’ One participant cited competing priorities in an overwhelmed healthcare system as to why there was not greater focus on LGBTQ+ identity: ‘in an NHS pressed on resources and time and energy it sometimes feels like yet another thing to have to worry about, and I know certain professionals just don’t see it as a priority.’ Time for continuing professional development was also highlighted ‘there are so many competing demands when it comes to providing good health care’. Such concerns around prioritisation were also highlighted in work by Ussher *et al.* and are clearly not unique to the UK healthcare system (34). However, there were notable absences from the list of structural biases in our study due to the free nature of the NHS including those related to insurance, and patient rooms, where the NHS has recently published clear guidance (62).

Several participants suggested a way to make the topic of disclosure easier to broach could be to have questions regarding SO and GI as standard on registration forms with an option to opt out from answering: ‘if it was a standard on the registration form, how do you identify? that would automatically raise it as everyone gets asked.’ This normalisation has previously been used by HCPs in the USA (36).Our participants took this one step further and suggested the inclusion of these questions in a commonly used health assessment tool used in their long term follow up clinics: ‘because they fill that in, they’re already on the wavelength that we will be talking about more than just their cancer.’ Another participant reflected that these questions could be asked indirectly through a psychosocial risk assessment tool used in the UK, the HEADSSS (Home, Education & Employment, Activities, Drugs/Drinking, Sex Self-harm, depression & suicide, Safety) assessment (63): ‘I think there’s a HEADSSS questionnaire for teenagers that I’ve heard of and used in the past and maybe thinking about more in my consultations right at the beginning and that would bring up things about relationships and I guess will bring up SO.’ Facilitators of disclosure may be adapted to the tools and processes of specific healthcare systems.

#### 3.2.5 Participant Age and Development

There is a notable absence in the literature of the challenges in facilitating SO or GI disclosure across different age groups. However, one of our participants described discomfort in dealing with LGBTQ+ identity in young people stating that they were: ‘very conscious that we’re dealing with people whose identity is forming.’ Belief that one’s patients may be too young to fully identify as part of the LGBTQ+ community therefore proved a further barrier to enquiry and engaging with this topic. This underlying assumption may in fact be a reason that this topic arises so rarely in the literature on HCP attitudes, because a proportion assume that the younger age groups that they treat will not be questioning their SO or GI, or at least will not have settled on a particular identity, and so never enquire about it, and do not discover anything to the contrary.

#### 3.2.6 The Role of the Healthcare Team

Another novel finding was that participants in our study particularly highlighted the role of members of the multi-disciplinary team (MDT) leading on a patient’s care in leading by example in respecting LGBTQ+ identity and encouraging disclosure conversations: ‘there is something about leadership, leading that care, introducing those questions (on SO/GI) I think that spreads … when it comes to creating cultural shift.’ Another participant felt secure to adopt a consultation style facilitating disclosure through being friendly and informal because they were ‘very well supported in my approach from my lead.’ While a supportive healthcare community has been shown to facilitate disclosure by the patient (55, 58), it appears that it also facilitates comfort with enquiry by the HCP.

### 3.3 Parental-Carer and Patient Dynamic

Many of our themes were those that appeared to influence LGBTQ+ patient care beyond simply disclosure. One such was the carer-patient dynamic, which takes on a unique form in young people where that carer is often a parental figure rather than a partner or child as is frequently the case in older adults. There is extensive literature on the influence of parents on the overall health and wellbeing of LGBTQ+ young people (64). Family acceptance of LGBTQ+ identity is associated with improved mental and physical health (63) and individual family dynamics are known to be affected by cultural background and whether a patient is ‘out’. HCPs in the study by Banerjee *et al.* also noted more strained communication in cancer care for young people who were not out to carers, parents or family (36).

The carer and patient dynamics were found to impact LGBTQ+ patient care both positively and negatively depending on the individual family dynamic. The patient’s carer could act as a barrier to HCPs asking more personal questions on SO and or GI. At times, HCPs felt the focus of the consult was addressing the parents’ questions and the patient did not engage. One participant described a situation of the lack of open dialogue between carers and patients regarding their cancer diagnosis: ’we still have parents who don’t tell their child that they’ve had cancer’. This dynamic was uncomfortable for the HCP and this environment does not set the tone for enquiry, disclosure or prioritisation of the patient’s needs.

However, the role of parents as potential advocates for their child’s LGBTQ+ identity was noted: ‘we had a (patient) who came in with his mum. His mum told the front desk that he wanted to be named by a male name and that was his identity.’ Support from the parent encouraged the HCP looking after this patient to ensure documentation reflected his GI and new name. Acceptance from the carer, can make this topic easier for HCPs to broach and discuss openly.

Another consideration raised was the importance of the HCP to build a trusting relationship with the carer to be able to look after their child: ‘respect and trusting relationships are three-way thing. It’s not just with the young person that’s with their parents and carers as well.’ This adds a unique complexity to caring for LGBTQ+ young people with cancer. There was a suggestion that if a parent is not comfortable with their child having an LGBTQ+ identity, then visual materials that display clinician comfort or what may be perceived as encouragement of LGBTQ+ identities may harm the clinician’s relationship with the parent: ‘if you’re a parent, you wouldn’t want to see things like that on the wall you have to take parents kind of concerns and feelings into consideration as well.’

The factors of being ‘out’ to parents and of culture/ethnic background noted in the general literature as being crucial in the parent-child dynamic (64) were also born out in our discussions with participants about this dynamic in their consultations: ‘if there’s a significant other that they’ve (the patient) not told their parents about, for example, which might be the case, that might come out.’ And ‘if there was a somebody from an ethnic minority, and they’re in a gay, lesbian relationship, which might not be so acceptable in their culture.’ The latter point also brings out the importance of intersectionality and how we need to consider the multiple factors that may affect someone’s experience of healthcare.

Another topic raised was the change in dynamic between patient and carer as there is less space for privacy once a patient is diagnosed with cancer: ‘when a young person particularly is diagnosed with cancer often you know they might be quite independent before, and then suddenly they’re in this situation where they’re having their parents more involved again’. HCPs may have a role to play in supporting patients to maintain independence at this time and LGBTQ+ identity may feature in this. They may require more specific training to do so.

### 3.4 The Patient as an Individual Outside of Their Cancer Diagnosis

Some of our participants recognised that teenagers/young adults may be going through more than their cancer treatment: ‘maybe the cancer is not the important thing at the moment or there’s other things going on in the background that are quite important to the patient, either less, more, or just as important as their diagnosis’. This may include dynamics with parents or family in relation to ‘coming out’.

Unlike adults whose carers are frequently also partners, children and teenagers are unlikely to have a partner present within the consultation. Fish *et al.* recognised partners as ‘a potential salutogenic resource’ for disclosure of SO in their interviews with adult LGB oncology patients (45). The lack of this aid to disclosure and advocacy in the room can be partially overcome by enquiry about their wider lives, including inquiry around partners.

Some HCPs also emphasised the importance of understanding the wider context of their patients’ lives for better overall patient care. One participant that did this as part of their consultation felt ‘it seemed quite natural for people to talk about their health care in the context of their life more broadly.’ Work by Fish and colleagues (45) interviewing LGB cancer patients found that disclosure of SO was driven by authenticity achieved by ‘a positive response to the disclosure of SO and a shared recognition by both patient and professional that the whole self is relevant to health.’

Given Rossman and colleagues (65) previously found that a major reason for non-disclosure by LGBTQ+ young people to HCPs was perceived lack of relevance to healthcare, this appreciation of the whole patient beyond their cancer may indeed facilitate greater disclosure as well as yielding other benefits.

### 3.5 Discussing Sex as Part of Cancer Care

Cancer diagnoses in young people may result in a delay in both the biological and social aspects of psychosexual development and education; its assessment is variable and clear consistent guidelines are lacking (66). LGBTQ+ young people report less satisfaction with this aspect of their oncology care than those who do not identity as LGBTQ+ (67).

However, the suggestion that you can talk about sex without discussing SO or GI was seen commonly throughout our interviews. Sexual activity tended to be discussed in a heteronormative form such as in discussions regarding contraception to avoid pregnancy and preserving fertility: ‘if you’re consenting for treatment and you’re talking about risks of getting pregnant.’ The interview sparked realisations from one HCP such as ‘that might make them feel uncomfortable.… talking in a way which clearly wouldn’t apply to their situation, if you’re talking about your husband and if you’re sexually active then it’s important you use contraception’ in reference to a patient in a same gender relationship.

Having appropriate tailored conversations around sexual behaviour may be particularly important in those with chronic health conditions as it has been linked to increased risky sexual behaviour (68).

When discussing a new weekly clinic which caters specifically for the holistic needs of the teenage and young adult patients, one HCP explained: ‘sexuality and fertility for sure is discussed there but I don’t know how easy or difficult it would be to discuss SO in that particular clinic’.

Russel et al. reported that LGBTQ+ cancer survivors reported less distress and concerns around infertility (69). This does not mean it does not deserve discussion but perhaps that it can be better balanced with the patient’s other psychosexual priorities.

It appears that, as noted in previous literature, appropriate education is lacking. One participant had attended a workshop about sex with cancer. She explained that it was: ‘about sex, not gender and it was fairly practical … it didn’t address anything specific about the different sexualities.’ Yet some HCP had still felt able to have these conversations with an LGBTQ+ young person ‘we had a conversation about sexuality … that might help sexual pleasure and playing with toys and things’ and that this yielded other benefits for the patients’ overall healthcare

Discussion of sex is of course another area of care where the patient-carer dynamic may be relevant: ‘it’s quite often difficult because you’re consenting patients, when often the parents are in the room, like about contraception … you have to be so sensitive because some people get really offended if you ask them if they are sexually active’, and links the importance of the appropriate setting for such discussions.

Patients also appear to be more likely to disclose LGBTQ+ identity if their cancer is related to their sexual or gynaecological health (70). Sensitive discussions around sex during cancer care provide a key opportunity to encourage disclosure of LGBTQ+ identity to then better tailor other information and management, and invite questions from the patient.

### 3.6 Lack of Confidence in Knowledge of LGBTQ+ Cancer Care

A number of studies have looked at LGBTQ healthcare knowledge across different HCPs within and outside oncology (29–37, 56, 71–76). Most recently a UK study of oncologists treating adults found that only 8% felt confident in their knowledge of the specific needs of this group (29). In the UK, the majority of oncologists treating children and teenagers are paediatricians and knowledge has also shown to be limited in this group (38). In a survey of US oncologists by Schabath *et al*, measures of confidence in knowledge fell after questions that tested specific LGBTQ+ healthcare knowledge had been answered, suggesting that studies such as these act to uncover educational blind spots (33).

Lack of confidence in knowledge on LGBTQ+ identities and healthcare was a common theme throughout the interviews. Most participants felt they lacked knowledge of LGBTQ+ cancer care and the importance of knowing your patient was part of the LGBTQ+ community: ‘I’m no expert, maybe it is more important that we do know.’

There were some areas of LGBTQ+ healthcare that HCPs felt were particular knowledge gaps. For example, how much to question their patients’ feelings regarding SO and GI: ‘this whole issue of emerging identity is very tricky’. This is a specific issue of concern in treating paediatric patients and has not been given focus in previous literature.

Based on the literature, HCPs are less knowledgeable and confident regarding trans and gender diverse patients (29, 33, 34, 77) as opposed to LGB healthcare. All interviewees in our study stated they did not have knowledge on this topic. Sutter *et al.* found this in part to stem from a relative lack of clinical experience with transgender patients (31). HCPs were also unaware as to when during their journey on questioning GI would a patient warrant a referral to an outside organisation such as the Gender Identity Clinic for an assessment.

Length of clinical experience was suggested as a barrier to accepting new education and improving confidence: ‘I have an assumption that the longer you’ve been doing this and the older you are the harder it becomes to stay in touch with more recent developments in what good health care looks like.’ However, this suggestion is in contrast to qualitative studies in this field. Berner et al. and Schabath et al. saw no significant effect of duration of experience in responses to their surveys on knowledge, attitudes and behaviours of oncologists treating LGBTQ+ patients in the UK and US respectively (29, 33). This is perhaps as these types of survey may attract greater numbers of professionals invested in the topic.

There was awareness of not treating members of the LGBTQ+ community as one homogenous group: ‘I think there are loads of nuances in terms of the needs of the community that often go unnoticed’ yet there was little discussion about the nuances of addressing LGBTQ+ identity across different age groups, perhaps highlighting a further ‘blind spot’.

Finally, however, some participants had little insight into their lack of knowledge of LGBTQ+ healthcare. Some of the most confident statements given by HCPs were that knowing a patient identified as LGBTQ+ would not change their medical management stating, ‘it wouldn’t impact on the treatment decisions.’ The underlying assumption here is that someone’s LGBTQ+ identity would not be directly relevant to their medical management, which is not the case (71, 78). Other quantitative and qualitative studies have also demonstrated cohorts of HCPs who continue to hold these views (34).

### 3.7 Knowledge of Appropriate Language

An increasing awareness and acceptance of different SOs and GIs has brought about terminologies and a change to language used to address patients, and to describe their identities and bodies. Use of appropriate language is key to cultural competence and humility in LGBTQ+ healthcare (79, 80).

Studies measuring knowledge, attitudes and behaviours of HCPs have focused less on knowledge and use of correct terminology. However, the commonly measured behaviour of enquiry on pronouns is low (29).

Knowledge of understanding the correct language to use with regard to LGBTQ+ identities was a theme throughout the data: ‘I don’t think I feel comfortable with those terminologies because I don’t quite understand some of the broader terms’, ‘I have to confess it was not that long ago I got something that said LGBTQ+ and I was like what is the Q and what is the +.’ This lack of knowledge included many aspects of language including pronouns, terminologies for identities and when to use neutral or gendered language.

Participants were aware of the importance of using the appropriate pronouns and appropriate name for trans young people and patients questioning their GI: ‘if a patient is just coming out as trans and they want to identify as a different sex with a different name to what their birth certificate name is written and their medical notes, then you know it’s discussed very openly so the team know how to address the patient.’ The use of gender neutral terms such as partner vs gender specific terms such as boyfriend/girlfriend was also highlighted by one participant: ‘I always use the term partners or partner.’

One participant cited a lack of consensus regarding different terminology as a barrier to knowledge and use of appropriate language: ‘it’s because there’s a lack of agreement … I know that some people even oppose the term LGBTQ+ and some people are using LGBTQI+, so you know, it’s very basics we can’t even agree on the language then having these conversations does feel impossible.’ Educational materials must therefore not only teach language and how to how to use it, but also how to stay up to date and manage mistakes. One strategy discussed was to follow the language used by the young person, ‘I very much rely on the language that young person uses.’

### 3.8 How Knowledge of LGBTQ+ Cancer Care Might Be Acquired

Participants also spoke about where they had acquired knowledge of LGBTQ+ healthcare and how they would fill gaps in their knowledge. None of the participants received specific training on LGBTQ+ health during their professional education: ‘I think this is something that in medical school … when I joined, it just wasn’t an open topic and people weren’t taught … how to support these patients. It’s probably an area that’s missing from my training.’ Some participants had attended a departmental teaching session on this topic which served to increase knowledge but also increase confidence to discuss this topic: ‘I think that just brought down all barriers to be able to talk about that between staff.’

The majority of participants said they would turn to self-education if there was something they didn’t know about LGBTQ+ health. At least half admitted they would need to go online to use google or social media to find LGBTQ+ friendly information for their patient: ‘I would basically start just looking on Google and social media.’ This presents a danger given the misinformation that can be present online from unreliable sources, and that transgender healthcare best practices can differ between countries.

Participants discussed acquiring knowledge through conversations amongst colleagues in order to increase one’s confidence to have these conversations with patients: ‘start these conversations professional to professional before they’re going to feel confident having those conversations professional with family.’

Others stated they would seek advice from colleagues or personal friends who identified as part of the LGBTQ+ community: ‘I have a lot of friends that identify as LGBTQ+ and so I would ask them and I know a lot of doctors as well that identify and you know I would just go and ask for support from a lot of reputable people that I very much trust and ask them how I could help.’ HCPs who had family members who were part of the LGBTQ+ community also drew on their own experiences: ‘I have got some personal experience … which is pertinent to my answers.’ However, in all of these cases, this relies on quality of the knowledge and experience of the person being approached. As the LGBTQ+ community is not a homogenous group, personal experience does not guarantee cultural humility, or indeed health expertise. While these methods are an adjunct to professional education and training, they are not a substitute for it.

Participants spoke about the experiential learning during consultations with LGBTQ+ patients: ‘I would continue to probably learn every time you know and build upon that’. This is of course an important aspect of continuing professional education but requires some baseline knowledge, and a degree of reflective practice. Indeed, one participant found the discussions from the interview for this study were a start to initiate reflection and how their practice could be changed to improve LGBTQ+ health: ‘having research forums like this and being able to sit and reflect and think about it probably makes it easier to think about how you do this in real time.’

### 3.9 The ‘Third Party’, as the Expert on the Topic of LGBTQ+ *Cancer*

A recurring theme in our interviews was the assumption that it was the responsibility of a ‘third party’ to be the expert in the topic of LGBTQ+ cancer rather than the individual themselves, as that person had more knowledge.

When HCPs were asked how they would manage a hypothetical scenario of a patient who was questioning their GI, the majority of participants stated they would include another member of the MDT: ‘I will obviously ask him if they want me to seek somebody who might be able to support them with that because I wouldn’t be best placed’ and ‘ensuring that I was well supported and had someone to turn to that had more experience would be really important.’ While it is good practice seek assistance from those with greater knowledge and experience, this should not be used as an excuse to not upskill oneself.

Specifically, oncologists felt their role was to focus on the medical management whilst the rest of the MDT would provide holistic care. One comment in regard to discussing SO and GI was: ‘that would come up in the holistic needs assessment. The CNS’ and ANP’s do that, we don’t, we tend to be focusing on the diagnosis and the treatment plan.’

Interestingly, whilst the oncologists would turn to other members of the MDT: ‘Our MDT have people within the team who are hopefully more knowledgeable in that area than me’, ‘these are very often issues that come out with our nurse specialist’, ‘there will probably be others in the team and psychologists in particular, who might have more insight into than me’, an allied health professional would seek support from the consultants: ‘I will follow it up in some way or another by speaking with a consultant’.

One participant expected staff wearing the NHS Rainbow badge to provide support: ‘having those (badges) within the trusts and particularly identifying people that you know have started to wear them very proudly they are the people you can turn to when you really do need advice on these sorts of issues and patients and how you could support them.’ As we have discussed, this may be an indicator of moral support but not expertise.

Some participants suggested a referral to psychology was important for a patient questioning their GI asking: ‘whether this was something they’d like to disclose with the psychology team who might have better training and how to help them with the process.’ Whilst many gender diverse individuals do seek psychological support, in some cases this may not be necessary and in others, not sufficient in terms of support.

While learning within the team is important, deferral to other professionals to explore topics specific to LGBTQ+ health, robs the individual clinician from valuable learning and disincentives them from educating themselves. This is an example of where a clinician attitude can have a direct impact on both knowledge and behaviour.

### 3.10 Visible LGBTQ+ Affirming Materials

Most participants were in agreement of the importance of visible LGBTQ+ affirming material in the healthcare setting as a visual symbol of support and safety. This included the NHS Rainbow Badge, rainbows lanyards and poster boards displaying LGBTQ+ colours/imagery and specific information.

Multiple studies in the UK and US have found the inclusion of LGBTQ+ affirming symbols in the healthcare environment to be welcomed by LGBTQ+ people of all ages as they facilitate disclosure and a feeling of acceptance to identity (45, 59, 81, 82). They have also been recommended by several best practice reviews on the topic (77, 83).

The NHS Rainbow Badge initiative was launched in 2018 at the Evelina Children’s hospital and is a popular visible LGBTQ+ symbol in UK healthcare (84). This badge has the NHS logo on the backdrop of the rainbow pride flag and has become a symbol of allyship throughout the NHS (85).

The knowledge of, and attitudes towards, the rainbow badges varied between participants. Some felt wearing them was a positive movement and a way to show support to members of the LGBTQ+ community: ’I think the rainbow badges and the rainbow lanyards have made it a topic of conversation’, others felt attempts at allyship needed to be more genuine: ’I think we’re a little bit guilty of talking the talk, but not walking the walk, it’s almost if I’m honest, feels a little bit tokenistic at the moment.’ Wearers of this badge are required to sign a pledge in order to wear one and so one would hope that it at least signifies a positive attitude of the HCP towards engaging with LGBTQ+ healthcare needs. However, no test of specific knowledge or ability to signpost to support is needed, and there is therefore a danger that patients could be met with misinformation.

This outward impression of knowledge on this topic was also felt by participants: ‘identifying people that you know have started wearing them very proudly, they are the people you can turn to when you really do need advice on these sorts of issues and patients and how you could support them’ while others recognised that wearing a badge does not necessarily mean knowledge on this topic: ‘the thing about wearing the badge. I can highlight for myself; I don’t know what their needs (trans or non-binary patients) would be.’

Healthcare institutions need to assess how ready its staff are to provide inclusive care, before using symbols which advertise it as inclusive (85).

## 4 Discussion

This study identified 10 key themes related to the delivery of LGBTQ+ cancer care for young people (**Table 1**). As highlighted, many of these echo findings of previous studies with both HCPs and patients, though the qualitative nature of this study allowed us to identify novel findings related to HCP knowledge, attitudes and behaviours, and the factors underlying them. Some of these such as the influence of the patient-parental carer dynamic on HCP attitudes were unique to the treatment of children and young people whereas others (how HCPs acquire LGBTQ+ knowledge, the expectation of a ‘third party’ to be the LGBTQ+ expert) have general relevance to wider LGBTQ+ healthcare.

The fact that disclosure of LGBTQ+ identity was a major theme within our work was unsurprising given it is a gateway to further tailoring of cancer care and that disclosure of LGBTQ+ identity has been shown to be associated with greater emotional wellbeing and satisfaction with cancer care (45, 57). HCPs felt comfortable for patients to disclose to them but tended not to initiate these discussions and suggest that ‘the patient will bring it up if it is important’. This fits with the ‘egalitarian’ approach in line with the work of Ussher et al. who suggest that HCPs may adopt one of three ‘positions’ to LGBTQ+ cancer care; anti-inclusive, pro-actively inclusive, or egalitarian, the latter being where LGBTQ+ identity is accepted but is not seen as a priority for enquiry as it does not represent a particular healthcare need (34). This approach may not be the most appropriate given the lower rate of disclosure of TYAs patients with cancer compared to older adults (34) despite the younger LGBTQ+ population having higher disclosure rates in general (23). Factors specific to the interaction with healthcare may mean patients do not recognise the relevance of this information to their healthcare, so are less likely to disclose in this context (86).

It is reassuring that many of the facilitators of, and barriers to, disclosure we identified had been highlighted in previous literature, adding weight to the evidence that informs education on training on this topic. A novel barrier identified was a concern around patient age and development when discussing LGBTQ+ identity, and this deserves focussed research and a greater education for all paediatric HCPs. Unsurprisingly, parental-carer/patient dynamic clearly influenced clinician attitudes treating patients, and this could be both positive and negative. We recommend more focused research into this area and how best to balance supporting parents and preserving the autonomy and identity of the young person.

We identified leadership within the healthcare team as a facilitator of disclosure, perhaps because it addressed culture of fear amongst HCPs, as they knew they had support in case of mistakes. As questions about LGBTQ+ identity are not currently asked as standard, HCPs feared being seen as making assumptions, causing offence and using the wrong language. Although some of these specific fears have been highlighted in the literature (46, 48), they may remain ‘hidden’ by the findings of apparent HCP ‘comfort’ in treating LGBTQ+ patients that is seen in quantitative studies. Of course, patients may also fear to disclose due to anticipated discrimination and our findings highlight the need to create psychological safety (87) for both patient and HCP to facilitate disclosure. Education and training would also be greatly improved by explicitly tackling the explicit fears and difficult situation discussed in our study and others (34).

A plethora of studies have shown a lack of LGBTQ+ specific education across both oncology and paediatrics (29, 31–34, 36, 38, 56, 71, 72) and young people describe a lack of LGBT-tailored knowledge/support when accessing healthcare (21). We found specific lack of knowledge of, and confidence in using, language related to LGBTQ+ care. This may explain some of the poor performance measures of related behaviours in previous studies (29, 30, 32, 34, 36, 88) and for cases where clinicians in such studies felt less confident or comfortable. Adequate education in LGBTQ+ cancer care is clearly not being delivered through current undergraduate or postgraduate education (29, 33). Our study was able to uncover where HCPs were currently seeking information, such as through social media or trusted colleagues. These findings will enable us to target how best to upskill the current workforce. Although our study was small, it appeared that allied HCPs placed LGBTQ+ identity higher on their consultation agenda, and it may be that the physician’s curriculum could be improved by drawing on the education of other HCPs.

We highlighted an interesting novel theme of HCPs expecting a ‘third party’ such as a fellow colleague, a colleague from the LGBTQ+ community or even a friend from the LGBTQ+ community to be an expert on this topic. If everyone is presuming someone else is the expert, this can result in a situation where nobody is self-educating. This attitude indicates that there may be a role for ‘LGBTQ+ care champions’ (89) within the healthcare setting to act as role models and to help direct colleagues towards appropriate sources of education and training. However, this does not negate the responsibility of the individual HCP to continuously learn and upskill themselves in areas of health inequality.

Participants also looked to patients as the educators on LGBTQ+ identity. Whilst taking each patient experience as a learning experience is positive, relying on this as the sole method of education may result in errors in communication particularly with the first few consultations (and beyond if they do not have the correct feedback). This has important implications as if poor quality care is experienced by patients, it may increase their reluctance to disclose in future consults. It may also provide an inaccurate source of specific medical knowledge depending on sources that patients have used to educate themselves on their healthcare (75). Finally, it places an unnecessary burden on the young person with cancer, who is already navigating the challenges of their diagnosis and identity (7).

Overall, the lack of HCP knowledge on this topic highlights the importance of training to incorporate more than the medical context. Learning and working through a biopsychosocial model (a model of health and illness which reflects the need to consider the complex interaction of biological factors, psychological factors and social factors when understanding and managing a patient’s health) will hopefully give HCPs the confidence to practice their professions through a holistic lens. New initiatives such as the “Cancer in LGBTQ+ Populations’ chapter in the forthcoming ESMO-ASCO curriculum will help to reinforce that this knowledge is not ‘optional’, and should be an area of learning sought by those looking after teenage patients as well.

### A New Framework: The Cycle of Influence for HCP-Patient Interactions in LGBTQ+ Cancer Care

As authors, we sought to create a framework on which to hang our findings and make recommendations to improve cancer care for LGBTQ+ young people. Much of the work investigating the HCP role in LGBTQ+ healthcare has taken the role of the Knowledge-Attitude-Practice (KAP) Survey, originally developed to study anthropological behaviours such as family planning (90). Studies using this method tend to assume the linear relationship that knowledge affects attitudes which affect practices/behaviours (91). However, others have noted the reflexive relationship between behaviours and capabilities (including knowledge and training) as well as the ability for those capabilities to act *via* motivations and attitudes (92). Banerjee *et al.* noted the ability of increased knowledge of LGBTQ+ patients’ health needs with more positive attitudes and open-communication behaviours (36).

In our study, we saw examples of the interrelatedness of these aspects in our interviews. Most clearly, we also saw the influence of knowledge on attitudes: “certainly by our TYA ANP’s who are very tuned into this. They would engineer conversation … so that the patient can discuss it” (on discussion of GI/SO). Further, a key barrier to enquiry about LGBTQ+ identity was a lack of awareness of its relevance to the patient’s healthcare and increased knowledge appeared to raise its priority in the HCP agenda.

We also saw the effect of attitudes on behaviours around discussion of LGBTQ+ health: ”I think the attitudes are massively changed, and I assume the knock-on effect is that it makes people feel more comfortable to talk about it too” and the ability of knowledge to change behaviour *via* a shift in attitudes: “they did it as a really, really amazing interactive kind of quiz discussion/teaching session, and I think that that just brought down all barriers to be able to talk about that between staff because it was something that was just became very comfortable following that.” The ability of personal and organisation behaviours to change attitudes directly was also noted: ”having boards, having the rainbow badges and lanyards, and just having it as something that is not a taboo to talk about, just something that is easy to discuss.”

Consultation behaviours that involved SO and GI enquiry were also able to bring about increased knowledge, and reinforce the behaviour: “I think you gain a lot of knowledge from young people, so you know I do feel quite happy to facilitate those sorts of conversations and I would continue to probably learn every time”. Knowledge may also directly influence behaviour e.g., in knowing the correct language to use with a patient.

**Figure 1:**
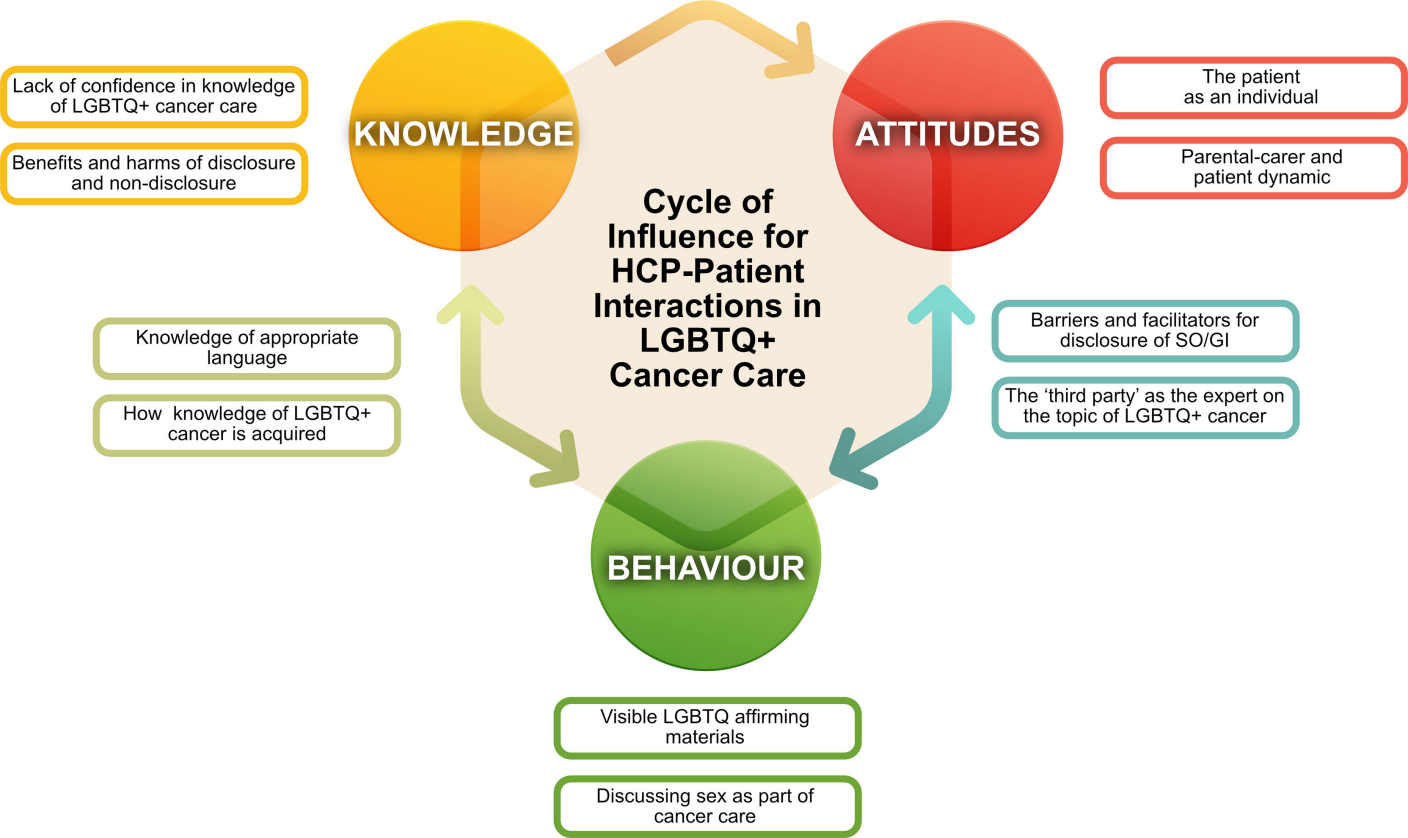
Cycle of Influence for HCP-Patient Interactions in LGBTQ+ Cancer Care. This framework describes how knowledge, attitudes and behaviours of healthcare professionals (HCPs) may interact and provides a tool from which to plan interventions for HCP education and organisational change.

Thus, we posit a highly reinforcing relationship of knowledge, attitudes and behaviours of HCPs in LGBTQ+ cancer care where influences may be cyclical and reciprocal (**Figure 1**). We also note some redundancy in that, for example, a positive attitude can be present without specific knowledge; ‘I don’t think you have to be an expert on this I think you just have to be open and sensitive’ but that the most effective behavioural change might come from working through this cycle: ‘I couldn’t say yes. I understand what they need … I would respect their decision, but I can’t say that I would have any insight in how to manage other than to use the pronouns that they’ve requested.’

The authors felt that our themes could be mapped to this framework directly such that 6 fell strictly under knowledge, attitudes or behaviours whilst 4 spanned the transitions (**Figure 1**). For example, barriers and facilitators of disclosure could be both attitudinal and behavioural, and frequently an interrelation of the two (although a major facilitator was knowledge of relevance of identity to healthcare). Knowledge of the correct language to use could directly influence communication behaviours. The authors suggest that future efforts to improve LGBTQ+ cancer care *via* HCP education should consider this so-called ‘Cycle of Influence for HCP-Patient Interactions in LGBTQ+ Cancer Care’ (**Figure 1**).

### Recommendations

We suggest that our framework, if utilised along with other published tools (92) could stimulate a ‘feed forward’ process whereby HCPs upskill in a self-driven way. It may be incorporated into educational initiatives or used to review existing local practice.

Given the dearth of knowledge we observed, we recommend basic improvements with postgraduate clinician education on a number of topics (**Table 2**). There also specific behaviours of individual HCPs (**Table 3**) and organisations (**Table 4**) which could facilitate increased disclosure of LGBTQ+ identity and improved care.

**Table 2:** List of topics recommended to improve postgraduate education for on LGBTQ+ health and cancer care for healthcare professionals.

• LGBTQ+ terminology and appropriate language	
• Why, when and how to facilitate disclosure of SO and GI
• Intersection of gender-affirming and cancer care
• Sex during cancer treatment

(SO – sexual orientation, GI – gender identity).

**Table 3:** Individual practice points for improving cancer care for LGBTQ+ youth.

• Ensure appropriate space for consultations. • Ensure enough time for consultations. If not possible organise a follow up meeting. • Aim for appropriate members of the MDT to be present in the consultation • Enquire with CYP if they would like their carers present during the consultation. • Offer one-on-one time with the CYP without their cares. • Explain confidentiality to the CYP and abide to this when possible. • Provide the CYP with reasoning as to why questions on LGBTQ+ identity may improve their care. • Encourage the HCP leading in the CYP’s care to enquire about SO/GI and to lead by example. • Increase dialogue amongst colleagues regarding LGBTQ+ health. • Increase use of a psychosocial risk assessment tools to assist in asking question regarding SO and GI. • Discuss sex and contraception in a non-heteronormative way.

(CYP - Children/Young Person, MDT - Multidisciplinary team, SO – sexual orientation, GI – gender identity).

**Table 4:** Recommended changes at the organisational level to bring lasting change for LGBTQ+ health.

LGBTQ+ affirming materials	• NHS Rainbow badge • Name badges stating one’s pronouns • LGBTQ+ supportive posters (explaining the importance of disclosure) • TYA leaflets with same sex couples
Registration forms with gender neutral language including appropriate options SO, GI and trans status	
Appointed LGBTQ+ lead or ‘champion’ who undertakes regular training and facilitates education of others.	

(TYA, Teenage and Young Adult, SO – sexual orientation, GI – gender identity).

As HCPs appreciated that ‘there may be someone more knowledgeable on this topic than them’, each hospital speciality could have an appointed dedicated LGBTQ+ lead or ‘champion’ who needs to undertake regular training to stay up-to-date and supports education of others. This practice has been successfully employed elsewhere (89). This can act to change organisational culture and influence both knowledge and attitudes, but care must be taken that it does not provide an excuse for individual HCPs not to self-educate.

### Strengths and Limitations

To our knowledge, this is the only qualitative study in the UK addressing HCPs knowledge, attitudes and behaviours when treating LGBTQ+ young people with cancer. Its UK specificity means its findings and recommendations are directly applicable to the workings of the NHS. We uncover novel themes in this area that might underlie some of the trends in knowledge, attitudes and behaviours seen in other studies (36).

We acknowledge several limitations to this study including its single-centre nature. Three interviewees had attended a recent education session which may have influenced responses. HCPs with more interest in changing LGBTQ+ cancer health may have been biased to participate. We had difficulty in recruiting male participants in a predominantly female paediatric oncology department. Interviews being conducted by a researcher visiting from outside the organisation may have led to both increased comfort of participants and reluctance to disclose some views.

To address these limitations, this work will be extended to gain a broad national picture with a UK-wide survey which developed in conjunction with the findings from this study and previous literature (29). We will use this to gather further evidence for our themes, suggested framework and recommendations.

## 5 Conclusions

Paediatricians are often the first health-care contacts for LGBTQ+ adolescents who are developing their sexual and gender identities therefore they have the chance to make a difference of their experience of healthcare.

Our work pointed to disclosure as a key starting point to ensure this topic is more commonly discussed in healthcare. We found a feed-forward relationship to improving HCP knowledge, attitudes and behaviours related to LGBTQ+ healthcare which we term the ‘Cycle of Influence for HCP-Patient Interactions in LGBTQ+ Cancer Care’. We suggest that interventions with the greatest impact on patient care are those spanning the domains of these framework, addressing psychological safety and impacting the organisation as well as the individual HCP. We look forward to its utilisation for improvements in NHS services and clinician education in the UK and beyond.

## Data Availability Statement

Raw interview transcripts and quotes will not be made available but lists of raw codes will be made available on request. Requests to access the datasets should be directed to TG, younglgbtqcancerstudy@gmail.com.

## Ethics Statement

The studies involving human participants were reviewed and approved by Royal Marsden NHS Foundation Trust and the Institute of Cancer Research Ethics committee (Ref No: SE 1132).The participants provided their written informed consent to participate in this study.

## Author Contributions

TG –Ethics application, patient and public involvement, conducting of interviews, interview transcription, interview coding, thematic analysis, reviewing of framework, manuscript drafting and reviewing; AB – Interview coding, thematic analysis, derivation of framework, manuscript drafting and reviewing; BP – Revision of themes, reviewing of framework, manuscript reviewing and editing;

DS – Revision of themes, reviewing of framework, manuscript reviewing and editing. All authors contributed to the article and approved the submitted version.

## Funding

TG is funded by a National Institute for Health Research (NIHR) Academic Clinical Fellowship. AB is funded by a Cancer Research UK Clinical Research Fellowship.

## Conflict of Interest

The authors declare that the research was conducted in the absence of any commercial or financial relationships that could be construed as a potential conflict of interest.

## Publisher’s Note

All claims expressed in this article are solely those of the authors and do not necessarily represent those of their affiliated organizations, or those of the publisher, the editors and the reviewers. Any product that may be evaluated in this article, or claim that may be made by its manufacturer, is not guaranteed or endorsed by the publisher.

## Abbreviations

AMB, Alison May Berner (author); EPR, Electronic Patient Record; GI, Gender identity; HEADSSS, Home, education, activities/employment, drugs, suicidality, sex, safety; HCP, Healthcare professional; HCPs, Healthcare professionals; HRT, Hormone replacement therapy; LGBTQ+, Lesbian, gay, bisexual, transgender, queer or questioning; MDT, Multi-disciplinary team; SO, Sexual orientation; TG, Tamsin Gannon (author); TYA, Teenagers and young adults.
